# Demonstration of hetero-gate-dielectric tunneling field-effect transistors (HG TFETs)

**DOI:** 10.1186/s40580-016-0073-y

**Published:** 2016-06-15

**Authors:** Woo Young Choi, Hyun Kook Lee

**Affiliations:** Department of Electronic Engineering, Sogang University, Seoul, 04107 Republic of Korea

**Keywords:** Tunneling field-effect transistors (TFETs), Metal–oxide–semiconductor field-effect transistors (MOSFETS)

## Abstract

The steady scaling-down of semiconductor device for improving performance has been the most important issue among researchers. Recently, as low-power consumption becomes one of the most important requirements, there have been many researches about novel devices for low-power consumption. Though scaling supply voltage is the most effective way for low-power consumption, performance degradation is occurred for metal–oxide–semiconductor field-effect transistors (MOSFETs) when supply voltage is reduced because subthreshold swing (SS) of MOSFETs cannot be lower than 60 mV/dec. Thus, in this thesis, hetero-gate-dielectric tunneling field-effect transistors (HG TFETs) are investigated as one of the most promising alternatives to MOSFETs. By replacing source-side gate insulator with a high-*k* material, HG TFETs show higher on-current, suppressed ambipolar current and lower SS than conventional TFETs. Device design optimization through simulation was performed and fabrication based on simulation demonstrated that performance of HG TFETs were better than that of conventional TFETs. Especially, enlargement of gate insulator thickness while etching gate insulator at the source side was improved by introducing HF vapor etch process. In addition, the proposed HG TFETs showed higher performance than our previous results by changing structure of sidewall spacer by high-*k* etching process.

## Background

The steady scaling-down of semiconductor device with rapid progress of fabrication technology facilitated high-integration, high-performance [1]. However, scaling-down resulted in short channel effects and power consumption increased exponentially [2, 3]. Recently, low power consumption becomes one of the most important requirements as scaling-down in semiconductor industry with the rapid growth of mobile market.

**Fig. 1:**
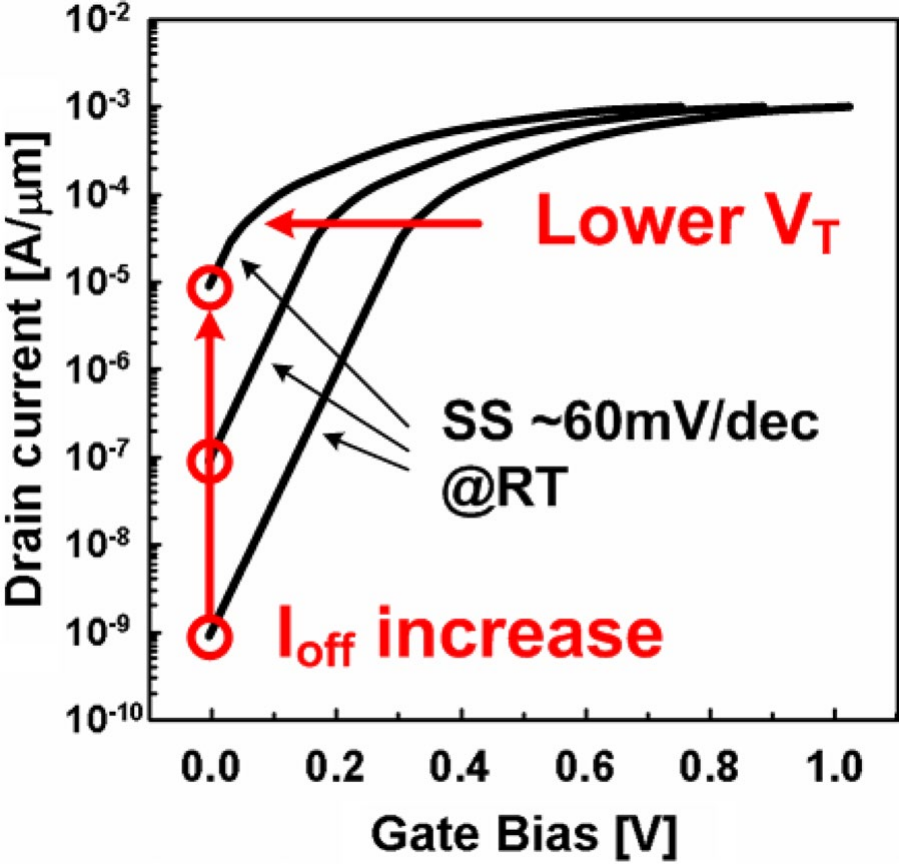
Threshold voltage scaling problem

The most efficient way to reduce power consumption is to scaling supply voltage (*V*_DD_) down which plays an important role in determining both standby and dynamic power consumptions. However, *V*_DD_ scaling of MOSFETs has been slower than device scaling because the downscaling of threshold voltage (*V*_T_) leads to a dramatic increase of off-current (*I*_off_) as described in Fig. 1 [4]. This is closely related to fundamental limit that subthreshold swing (SS) of MOSFETs cannot be lower than 60 mV/dec [5]. In the case of MOSFETs, carriers are injected from the source to the channel by thermionic emission mechanism. As the energy distribution of conduction electrons in the source follows the Fermi–Dirac distribution, electrons injected by increasing gate voltage (*V*_G_) also follow the Fermi–Dirac distribution which limits minimal SS around 60 mV/dec at room temperature.

Thus, many novel devices have been proposed recently to overcome fundamental limit. They include impact-ionization MOS devices [5, 6], nano-electro-mechanical FETs [7], and tunneling field-effect transistor (TFET) [8–23]. Among them, a TFET is considered one of the most promising candidates for ultra-low-power semiconductor device. TFETs show low *I*_off_ and sub-60 mV/dec SS at room temperature because electron flows are controlled by band-to-band tunneling mechanism. In addition, TFETs are less influenced by short channel effects than MOSFETs [14, 15] and complementary metal-oxide semiconductor (CMOS) process compatible. On the other hand, TFETs have disadvantages such as lower on current (*I*_on_) and ambipolar behavior [16, 17]. To overcome these problems, many studies have been reported by introducing various materials and device structures [17–23].

In this thesis, hetero-gate-dielectric tunneling field-effect transistors (HG TFETs) are investigated. HG TFETs show higher *I*_on_, lower ambipolar current (*I*_amb_) and smaller SS than conventional TFETs by replacing source-side gate insulator with high-*k* materials [18]. First, the theoretical background of TFETs and device concepts of HG TFETs will be covered. In addition, HG TFET design was optimized and improved through the simulation. As a result, HG TFETs showed higher performance than that of conventional TFETs. To improve the performance of HG TFETs, improved fabrication methods were proposed. Etching the gate insulator at the source side by using HF vapor improved enlargement of etched gate insulator thickness. In addition, structure of sidewall spacers was changed to remove the high-*k* layer on the source region by high-*k* etching process. This solved the problem that tunneling barrier width was increased by fringe field. After the overall process flow for the fabricating HG TFETs using standard CMOS process was introduced, electrical characteristic results of fabricated device demonstrated the simulation results. Proposed HG TFETs showed higher performance than our previous results. As a result, it is concluded that HG TFETs are promising to be used for highly energy efficient ICs.

## Theoretical studies

### Basic operations of TFETs

**Fig. 2:**
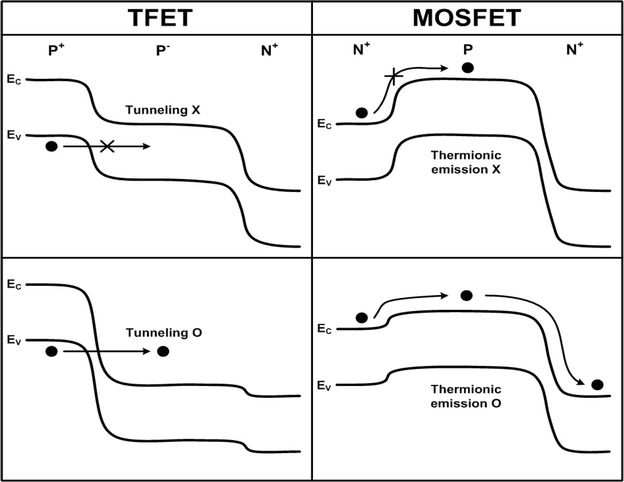
Energy bands of the TFET and the MOSFET during operation

Compared to MOSFETs, basic structure of TFETs is a gated p–i–n diode as shown in Fig. 2. Band-to-band tunneling mechanism is used as a carrier injection of TFETs instead of thermionic emission. Different operation mechanism between MOSFETs and TFETs comes from the asymmetric doping profile of source and drain of TFETs. In n-channel TFETs, the p^+^ source is grounded and the n^+^ drain is positively biased. In the off-state, TFETs resemble a reverse biased p–i–n diode and tunneling barrier width (*W*_tun_) between valence band of the source and conduction band of the channel is thick which make extremely low *I*_off_ flow. In the case of MOSFETs, electron injection from the source to the channel is hard because of high energy barrier between the source and the channel. In the on-state, when a positive gate bias induces strong band bending of channel and *W*_tun_ is narrowed, the valence band electrons from the source region tunnel through the barrier into the conduction band in the channel region. Thus, the TFET shows very sharp on–off transition and SS value of TFETs is not subjected to 60 mV/dec thermal limit like MOSFETs. These characteristics lead a TFET as one of the most promising candidates for low-power device. Despite those advantages, TFETs have several disadvantages to figure out. Because of high tunneling resistance, *I*_on_ of TFETs is much lower than that of MOSFETs and ambipolar behavior of TFETs increases leakage current [8]. To improve performance of TFETs, various techniques have been proposed. Since *I*_on_ of TFETs is determined by *W*_tun_ and electric field at the tunneling junction, introducing high-*k* materials as a gate insulator, narrow bandgap materials and novel device structures were shown. However, using high-*k* materials as a gate insulator increases *I*_amb_ by ambipolar behavior as well as *I*_on_ [14].

### Characteristics of HG TFETs

**Fig. 3:**
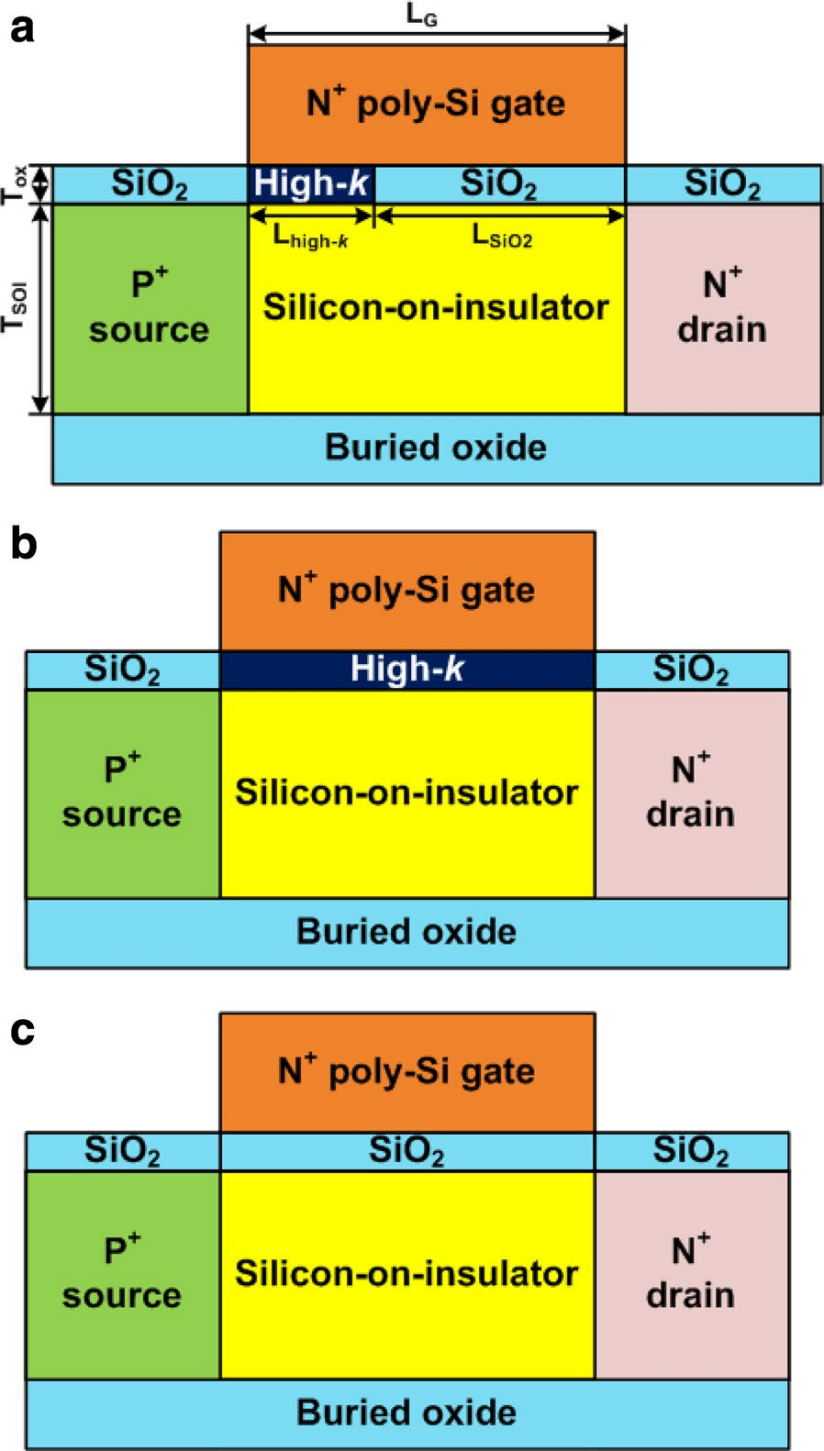
Schematics of an **a** HG, **b** high-*k*-only and **c** SiO_2_-only TFET

**Fig. 4:**
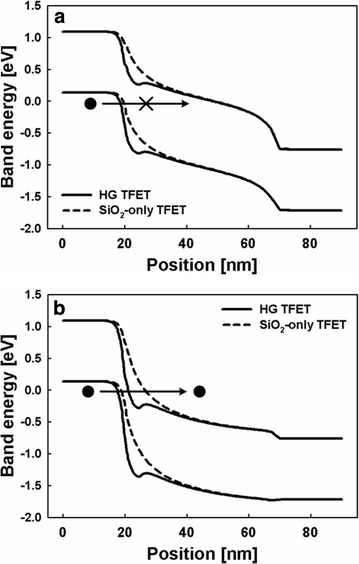
Energy band diagrams of HG and SiO_2_-only TFETs at **a** off- and **b** on-state

HG TFETs are proposed for higher *I*_on_, lower *I*_amb_, and smaller SS. In this study, HG TFETs will be compared with two kinds of conventional TFETs, high-*k*-only and SiO_2_-only TFETs as shown in Fig. 3. High-*k*-only TFETs use only high-*k* dielectric as gate insulator and SiO_2_-only TFETs use only silicon oxide (SiO_2_) as a gate insulator. The HG TFET is composed of different gate dielectric materials at the source and drain sides. A high-*k* material is only partially located at the source side and this leads to the particular energy band structure as shown in Fig. 4. HG TFETs show a local minimum of the conduction band edge (*E*_c_) due to relative permittivity discrepancy between high-*k* dielectric and SiO_2_ layer. HG TFETs show more abrupt change from off to on-state because *W*_tun_ of HG TFETs abruptly narrows when a local minimum of *E*_c_ is aligned with the valence band edge (*E*_v_) of the source region.

**Fig. 5:**
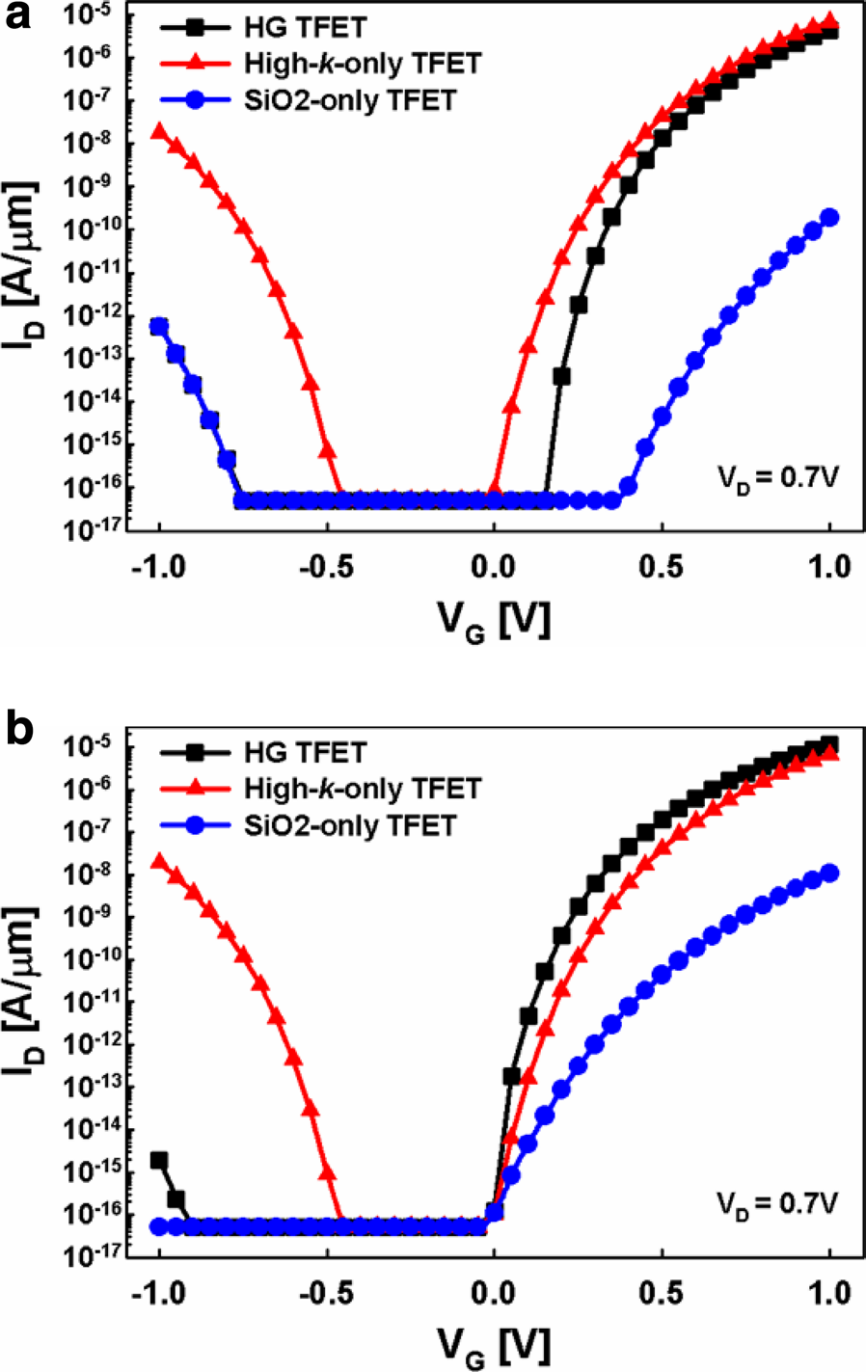
Transfer curves of the HG, high-*k*-only, and SiO_2_-only TFETs when **a** gate workfunction is 4.1 eV and **b** gate work function is adjusted that *I*_off_ is 0.1 fA at 0 V *V*_G_

To compare the performance of HG TFETs with high-*k*-only and SiO_2_-only TFETs, two-dimensional device simulation has been performed by using Silvaco ATLAS [24]. A nonlocal band-to-band tunneling model has been used. Band gap narrowing, Fermi statistics, Shockley–Read–Hall (SRH) recombination and Lombardi mobility models have been used in this simulation. Gate leakage current and quantum effect have been ignored. An abrupt source/drain junction profile has been assumed as shown in the previous works [18, 25]. Device parameters used in this simulation are summarized in Table 1. Figure 5a shows the transfer characteristics of HG, high-*k*-only and SiO_2_-only TEFTs that use n-type doped polysilicon gates. Optimized HG TFETs whose length of high-*k* material under the gate (*L*_high-*k*_) is 5 nm are used in this case. HG TFETs follows SiO_2_-only TFETs at low *V*_G_ because ambipolar behavior is determined by the drain-to-channel region overlapped by SiO_2_ layer. On the other hand, on-state of HG TFETs follow high-*k*-only TFETs because of high-*k* insulator locate at the source-to-channel region. For fair comparison, the gate workfunction is adjusted that *I*_off_ is 0.1 fA at 0 V *V*_G_ as shown in Fig. 5b. Because HG TFETs show higher *I*_on_ than high-*k*-only TFETs and have *I*_amb_ as low as SiO_2_-only TFETs, HG TFETs show lower SS than high-*k*-only and SiO_2_-only TFETs.

**Table 1:** Device parameters used for simulation

	HG TFET	High-*k*-only TFET	SiO_2_-only TFET
*L*_G_ (nm)	50	50	50
*t*_SOI_ (nm)	30	30	30
*t*_ins_ (nm)	2	2	2
Source/drain doping conc. (cm^−3^)	10^20^	10^20^	10^20^
Channel doping conc. (cm^−3^)	10^15^	10^15^	10^15^
*L*_high-k_ (nm)	5	50	X
*k* value of high-*k* dielectric	25	25	X

### Optimization of the device design

**Fig. 6:**
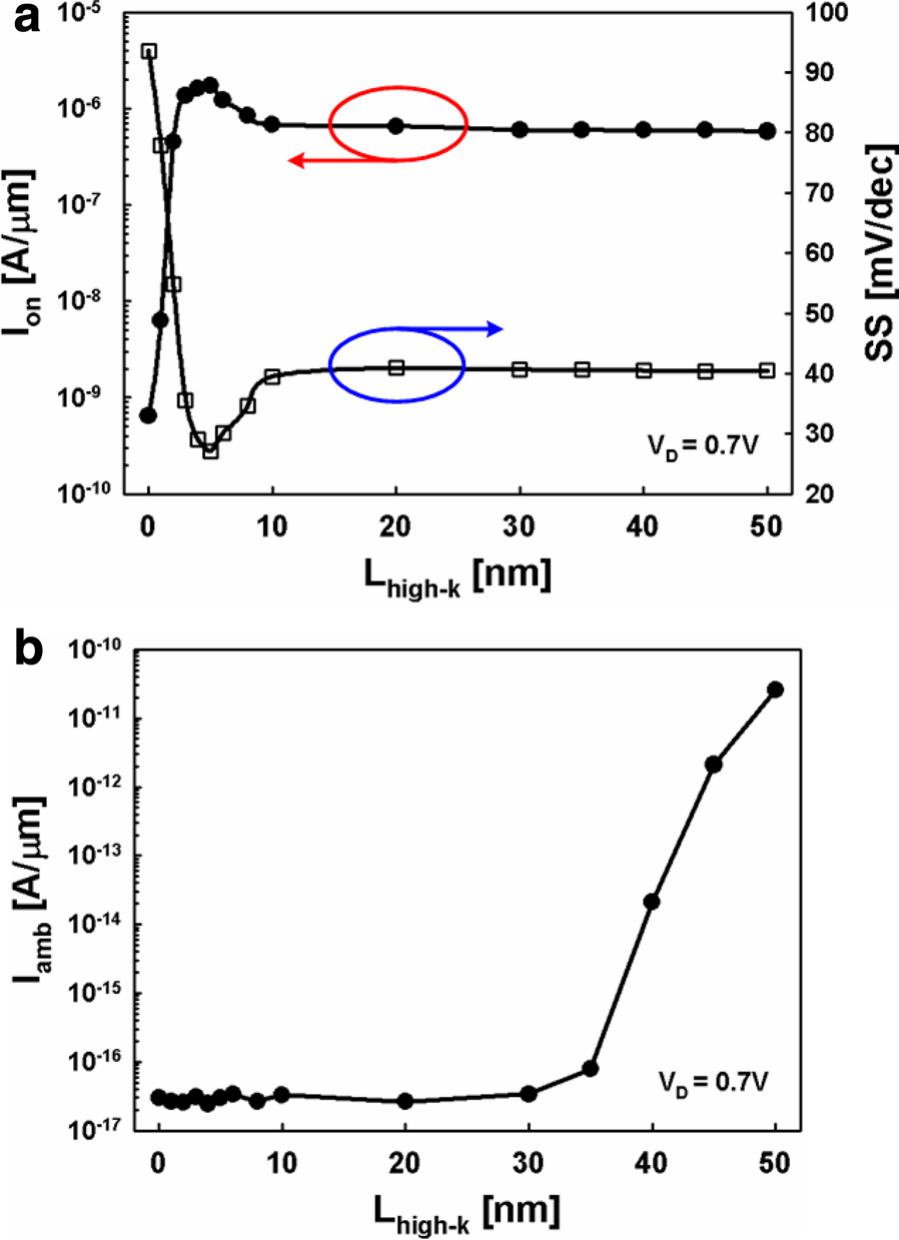
**a** *I*_on_ and SS and **b** I_amb_ of HG TFETs as a function of *L*_high-k_

To optimize the device design of HG TFETs, the design issues of HG TFETs such as *L*_high-*k*_ and silicon-on-insulator (SOI) layer thickness (*T*_SOI_) have been investigated. *I*_on_ is defined as drain current (*I*_D_) when both *V*_G_ and drain voltage (*V*_D_) are 0.7 V, *I*_amb_ is defined as *I*_D_ when *V*_G_ is −0.7 V and *V*_D_ is 0.7 V. SS is defined as an average slope when *I*_D_ is from 0.1 fA/μm to 0.1 nA/μm at *V*_D_ is 0.7 V. Figure 6a shows extracted *I*_on_ and SS as a function of *L*_high-*k*_. When *L*_high-*k*_ is optimized around 5 nm, HG TFET show ~40 % smaller SS and three times higher *I*_on_ than high-*k*-only TFETs. In addition, HG TFETs show ~70 % smaller SS and three orders of magnitude higher *I*_on_ than SiO_2_-only TFETs. Figure 6b shows extracted *I*_amb_ as a function of *L*_high-*k*_. Because *I*_amb_ is determined by ambipolar behavior at the drain side, *I*_amb_ abruptly decrease as *L*_high-*k*_ decreases. As a result, HG TFETs show six orders lower *I*_amb_ compared to high-*k*-only TFETs.

**Fig. 7:**
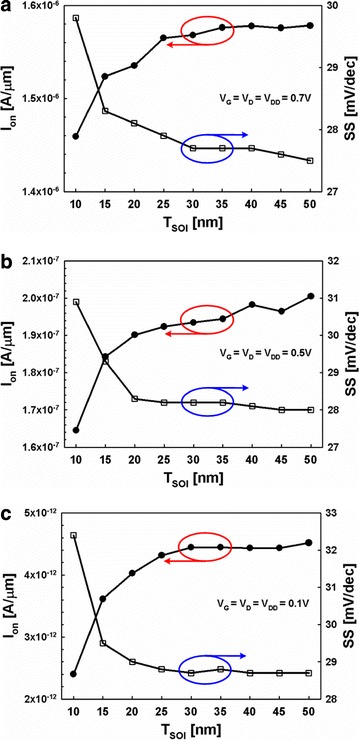
*I*_on_ and SS of HG TFETs with the variation of *T*_SOI_ for different *V*_DD_ conditions. **a** *V*_DD_ = 0.7 V, **b** *V*_DD_ = 0.5 V, **c** *V*_DD_ = 0.1 V

In addition, the effect of *T*_SOI_ has been discussed in terms of *I*_on_ and SS. Figure 7 shows extracted *I*_on_ and SS as a function of *T*_SOI_ for several different operating voltage (*V*_DD_). *I*_on_ is defined as *I*_D_ when both *V*_G_ and *V*_D_ are equal to *V*_DD_. SS is defined as same as before. *I*_on_ of HG TFETs show little change as *T*_SOI_ decreasing when *V*_DD_ is 0.7 V. However, *I*_on_ of HG TFETs tends to become lower as *T*_SOI_ decreases at low *V*_DD_ as shown in Fig. 7b, c. In addition, decreasing *T*_SOI_ makes the SS of HG TFETs larger regardless of *V*_DD_. It is because the performance of HG TFETs is mainly determined by the difference in the gate-to-channel coupling strength between channel regions overlapped by the high-*k* insulator and SiO_2_ layer and it decreases as *T*_SOI_ decreases. As a result, it is difficult to form a local minimum on the conduction band edge and performance of HG TFETs worsens as *T*_SOI_ decreases. To sum up, large *T*_SOI_ can be helpful to get higher *I*_on_ of HG TFETs at low *V*_DD_ and SS of HG TFETs increases as *T*_SOI_ decreases regardless of *V*_DD_ [26]. As a result, 30-nm *T*_SOI_ is selected for fabrication this time.

### Improvement in device design

Our previous work showed worse HG TFET performance than expected [27]. It was concluded that this result came from some factors: gradual doping profile, enlarged high-*k* dielectric thickness at the source side and sidewall spacer structures. All of these factors are related to the fabrication process and these have been investigated to improve the performance of HG TFETs.

First, abrupt doping profile at the tunneling junction is very important for TFETs because it determines *W*_tun_ and electric field which control the tunneling current. Doping profile which is especially overlapped by high-*k* material has an influence on HG TFETs because performance of HG TFETs is mostly determined by formation of a local minimum of the *E*_c_ at the tunneling junction [18]. As a result, abrupt doping profiles at the tunneling junction are suitable for higher *I*_on_ and lower SS. However, gradual doping profiles are applied to our HG TFETs because we used conventional RTA instead of advanced annealing method. Thus, fabrication conditions which control the doping profile should be optimized. In general, doping profiles at the tunneling junction are influenced by the spacer length (*L*_spacer_) and the RTA time (*T*_RTA_). *L*_spacer_ is the sum of an inner high-*k* spacer length and an outer low-*k* spacer length. To adopt the fabrication conditions, two-dimensional semiconductor process simulation and device simulation has been performed by using Silvaco ATHENA and ATLAS [24]. In the case of process simulation, some conditions were changed from the conditions used for device simulation. Abrupt doping profile is changed to gradual doping profile which is determined by *T*_RTA_.

Second, high-*k* dielectric partially located at the source side increase the gate-to-channel coupling strength and this leads to the particular energy band structure [18]. HG TFETs show lower SS and higher *I*_on_ because of a local minimum of the *E*_c_ at the tunneling region. Though the thickness of high-*k* dielectric should be equal to *T*_ox_, this is enlarged during etch process of SiO_2_ gate insulator. Thus, the difference of the gate-to-channel coupling strength between channel regions overlapped by the high-*k* dielectric and SiO_2_ decreased. It degrades the performance of HG TFETs and solution to this will be discussed in chapter 3.

**Fig. 8:**
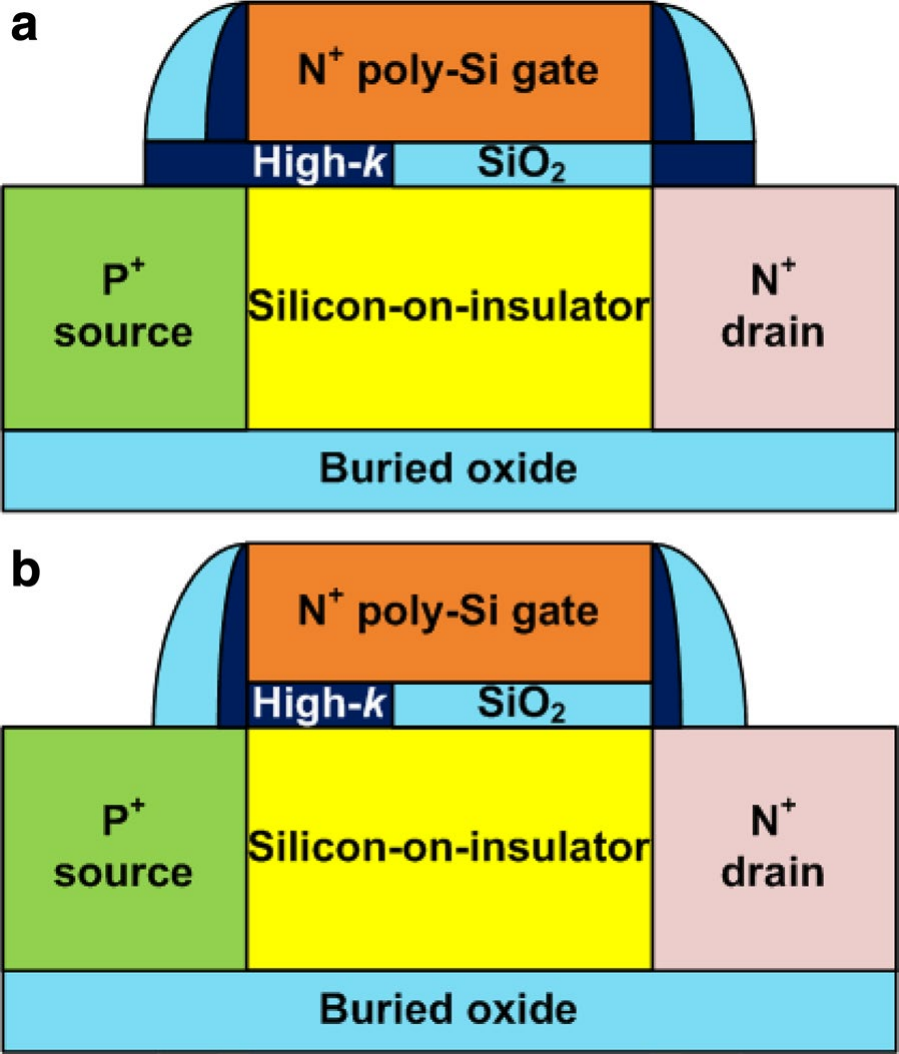
Structures of an **a** previous and **b** proposed HG TFET

Third, the sidewall spacer structure of our previous HG TFETs is problematic. Figure 8a shows the structure of our previous HG TFETs. Previous HG TFETs have gradual doping profiles and dual-*k* spacers which consist of 3-nm inner high-*k* spacers and 19-nm outer low-*k* spacers. High-*k* spacers are used to enhance the electric field around the tunneling junction and low-*k* spacers are used to control tunneling junctions [28, 29]. However, 3-nm high-*k* dielectric layers under the low-*k* spacers are the main factors which degrade the performance of HG TFETs. Because high-*k* dielectric layers are placed on the source regions, fringe field from gates increases as *V*_G_ increases. Accordingly, the energy bands of the source regions decrease as well as those of the channel regions. It makes *W*_tun_ larger and our previous HG TFET performance worse.

**Fig. 9:**
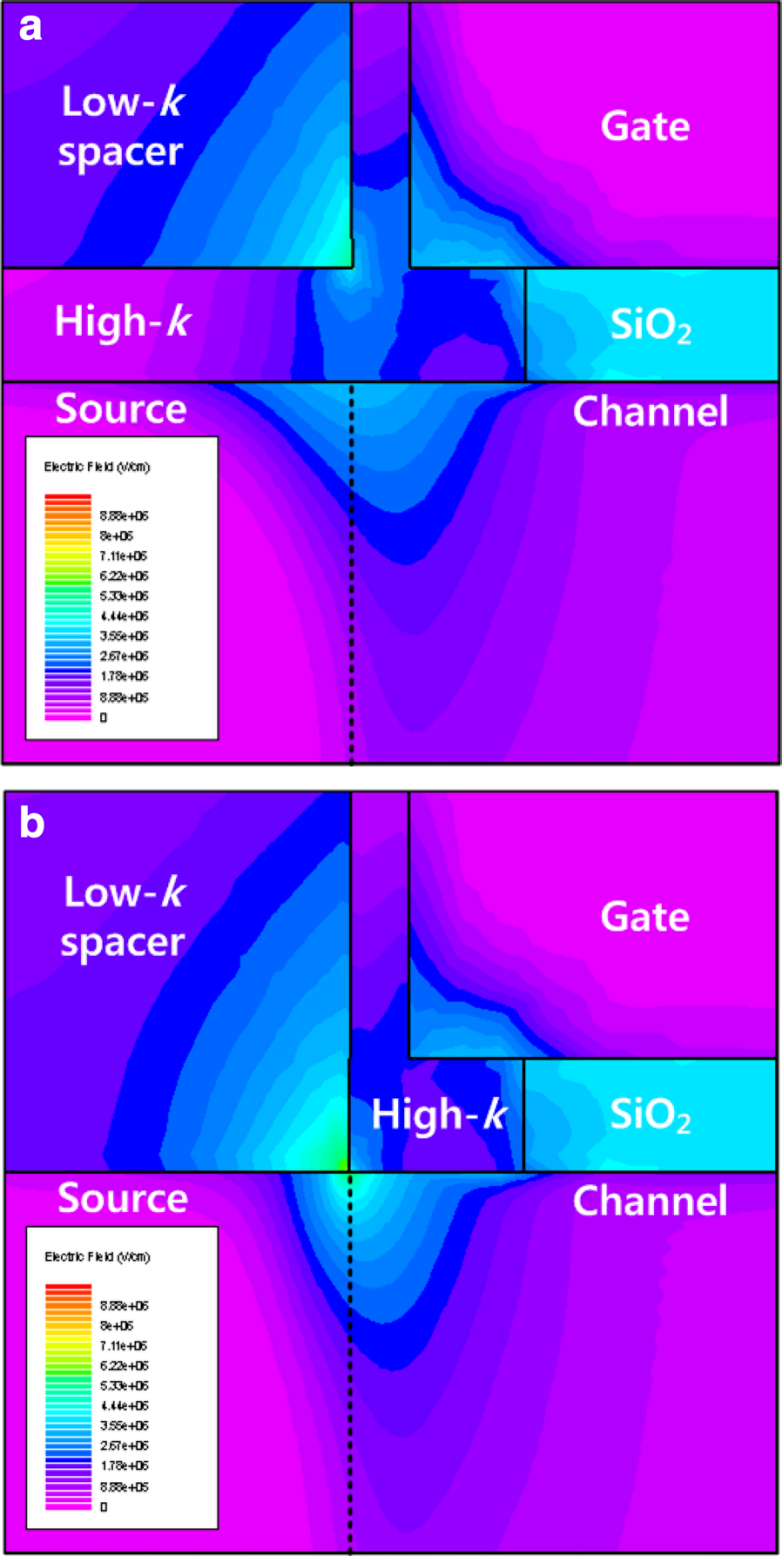
Fringe field through the spacer for **a** previous and **b** proposed HG TFETs

To enhance the performance of HG TFETs, the dependency of sidewall spacer structures on the performance has been examined. The structure of a dual-*k* spacer is improved as shown in Fig. 8b. A 3-nm high-*k* dielectric layer under the low-*k* spacer is removed and only a 3-nm inner high-*k* spacer is remained. To investigate the impact of the dual-*k* spacer structure on the performance of HG TFETs, fringe field around the tunneling region is compared as shown in Fig. 9. Inner high-*k* spacer increases the fringe field around the tunneling junction for both structures. Fringe field coupling through the inner high-*k* spacer decreases *W*_tun_ [28]. However, fringe fields are denser and higher near the junction in the proposed HG TFETs compared to the previous HG TFETs. In the case of proposed HG TFETs, fringe field is focused on the edge of the high-*k* spacer which is in contact with TEOS spacer. On the other hand, fringe field of the previous HG TFETs is low and spread because fringe field through the inner high-*k* spacer and high-*k* dielectric layer under the low-*k* spacer are balanced. Thus, gate potential is coupled over a large distance and this result in low current.

**Fig. 10:**
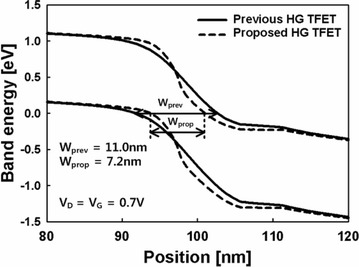
Energy band diagrams for the previous and proposed HG TFETs. *W*_prev_ and *W*_prop_ refer to the tunneling width for the previous and proposed HG TFETs

The impact of the fringe field coupling on the tunneling region is further illustrated by the band diagrams as shown in Fig. 10. The figure shows the band diagrams near the tunneling junction for *V*_G_ = *V*_D_ = 0.7 V. From the figure, *W*_tun_ of the previous and proposed HG TFETs have been compared each other. As mentioned before, *W*_tun_ of the previous HG TFETs is larger than that of proposed HG TFETs because fringe field through the high-*k* dielectric layer on the source region reduce the energy band of the source region.

**Fig. 11:**
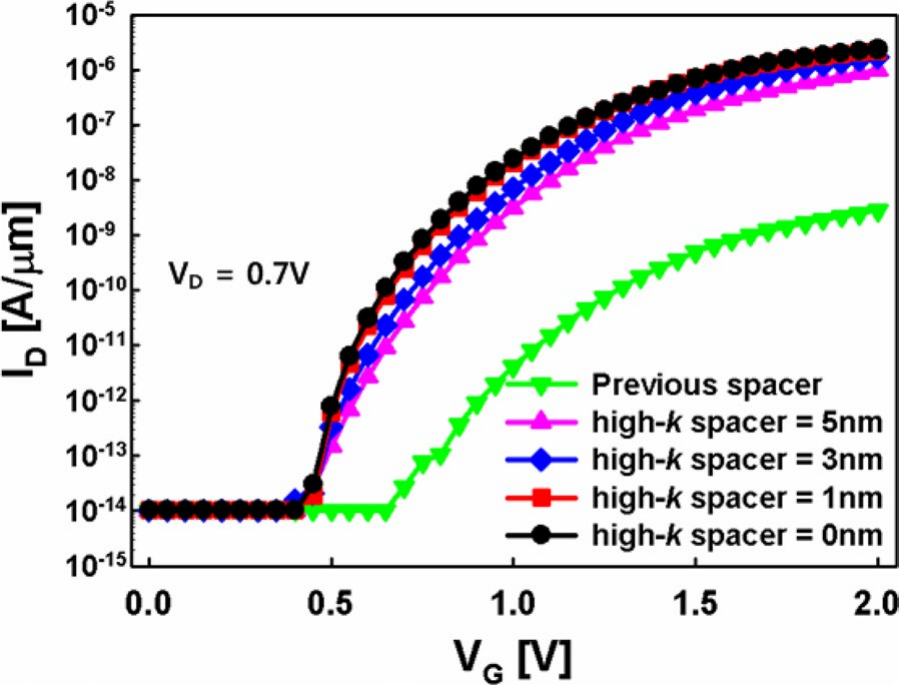
Effect of high-*k* spacer length variation on the transfer characteristics for the proposed HG TFETs which is compared to the previous HG TFETs

To optimize the design of HG TFETs, the effect of variation in the length of the high-*k* spacer on *I*_on_ has been investigated. Figure 11 shows the transfer characteristics of the proposed HG TFETs compared with the previous HG TFETs as the length of the high-*k* spacer varies from 0 to 5 nm. The length of an outer low-*k* spacer is fixed at 19 nm for all because of the tunneling junction. Performance degradation is more severe in the case of the previous HG TFETs because high-*k* dielectric on the source region increases the coupling between the gate and the source region. It is clear from the transfer characteristics in Fig. 11 that the device performance degrades with an increasing the length of the high-*k* spacer for the proposed HG TFETs. An increase in the length of the high-*k* spacer reduces the electric field from the gate because of the physical distance, thereby causing the degradation in the device performance.

**Fig. 12:**
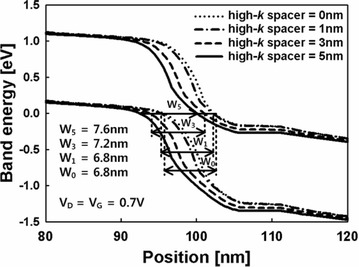
Energy band diagrams for proposed HG TFETs with the variation of high-*k* spacer lengths

Figure 12 shows the energy band diagrams of the proposed HG TFETs with various high-*k* spacer lengths at *V*_G_ = *V*_D_ = 0.7 V. *W*_tun_ was extracted from the point which shows the maximum electron tunneling rate. *W*_tun_ increases as the length of the high-*k* spacer increases which is consistent with the trend in the transfer characteristics. As a result, HG TFETs without an inner high-*k* spacer show the most improved performance. However, the length of high-*k* spacer in this study is 3 nm because of the fabrication issues and this will be covered in chapter 3.

**Fig. 13:**
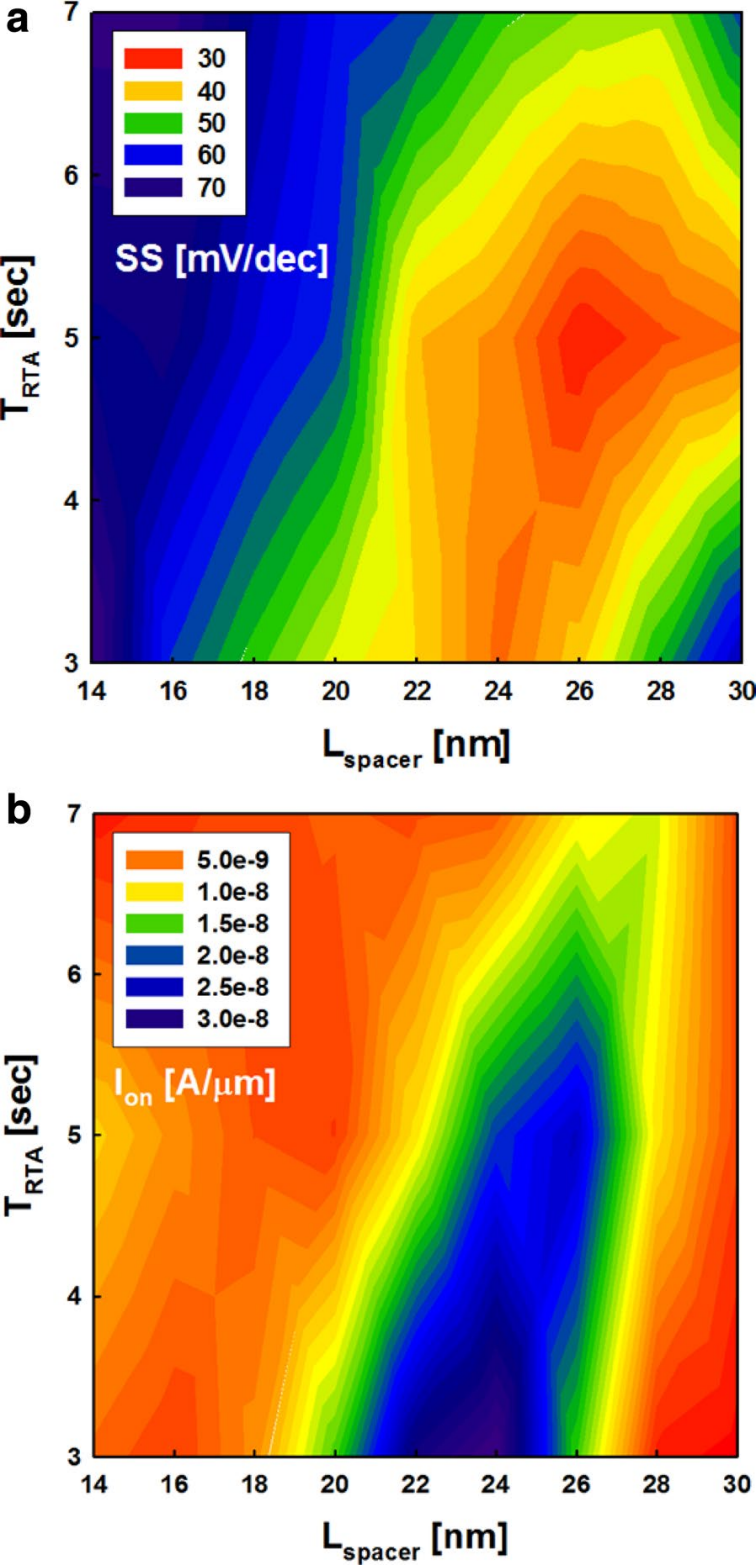
Contour plots of **a** SS and **b** *I*_on_ for the proposed HG TFETs with the variation of *T*_RTA_ and *L*_spacer_

To verify the fabrication condition for the proposed HG TFETs, the effects of *L*_spacer_ and *T*_RTA_ have been also discussed in terms of *I*_on_ and SS. Figure 13a shows extracted SS as a function of *L*_spacer_ and *T*_RTA_. When the device structure is formed by process simulation, SS is extracted from different range of *I*_D_ because leakage current level and average of SS are higher than those of device simulation. Thus, SS is defined as an average slope when *I*_D_ increases from 10 to 100 fA/μm. Regardless of *T*_RTA_, SS of HG TFETs becomes higher as *L*_spacer_ decreases because dopants of high concentration diffused from the source region are overlapped by high-*k* material. In this case, conduction band well becomes shallower because higher doping concentration makes *E*_c_ under the high-*k* material increases. On the other hands, SS becomes higher as *L*_spacer_ increases because of underlap between source and channel region. Similarly, when *L*_spacer_ is fixed, SS becomes higher as *T*_RTA_ decreases because of underlap structure. On the contrary, as *T*_RTA_ increases, conduction band well becomes shallower, which makes less abrupt transition between off- and on-state. When *T*_RTA_ is 3 s, minimum SS value is shown when *L*_spacer_ is 24 nm and optimum *L*_spacer_ increases as *T*_RTA_ increases.

Figure 13b shows extracted *I*_on_ as a function of *L*_spacer_ and *T*_RTA_. The turn-on voltage (*V*_turn-on_) is defined as *V*_G_ when *I*_D_ is 10 fA/μm. *I*_on_ is defined as *I*_D_ when *V*_D_ is 0.7 V and *V*_G_ is 0.7 V higher than *V*_turn-on_. *I*_on_ shows similar tendency observed in SS as a function of *L*_spacer_ and *T*_RTA_. Optimum *L*_spacer_ increases as *T*_RTA_ increases for the same reason. Tunneling current increases as electric field at the tunneling region increases and it is reversely exponential to *W*_tun_. Electric field is determined by the slope of the energy level in the band diagrams and *W*_tun_ is also strongly influenced by doping profiles. Mostly optimized *I*_on_ is shown when *T*_RTA_ is 3 s because more abrupt doping profile is formed as *T*_RTA_ decreases. When *T*_RTA_ is 3 s, optimum *L*_spacer_ is 24 nm as same as in the case of SS.

From the results of simulation, overlapped region between Fig. 13a, b is selected as the target for the fabrication condition. Finally, optimized *L*_spacer_ is 24 nm and *T*_RTA_ is 3 s. Though there is variability from the fabrication conditions, it would be within the margin of error because SS shows little change.

## Fabrication of HG TFETs and analysis

### Improvement in fabrication methods

As discussed in chapter 2, performance degradation was shown for previous HG TFETs and reasons are closely related to fabrication issues. Gradual doping profile is one of them and it is difficult to be improved because it needs advanced annealing equipments. However, there are solutions for enlarged high-*k* dielectric thickness at the source side and the structure of the sidewall spacer. Two key ideas have been introduced to enhance the performance of HG TFETs in this work.

**Fig. 14:**
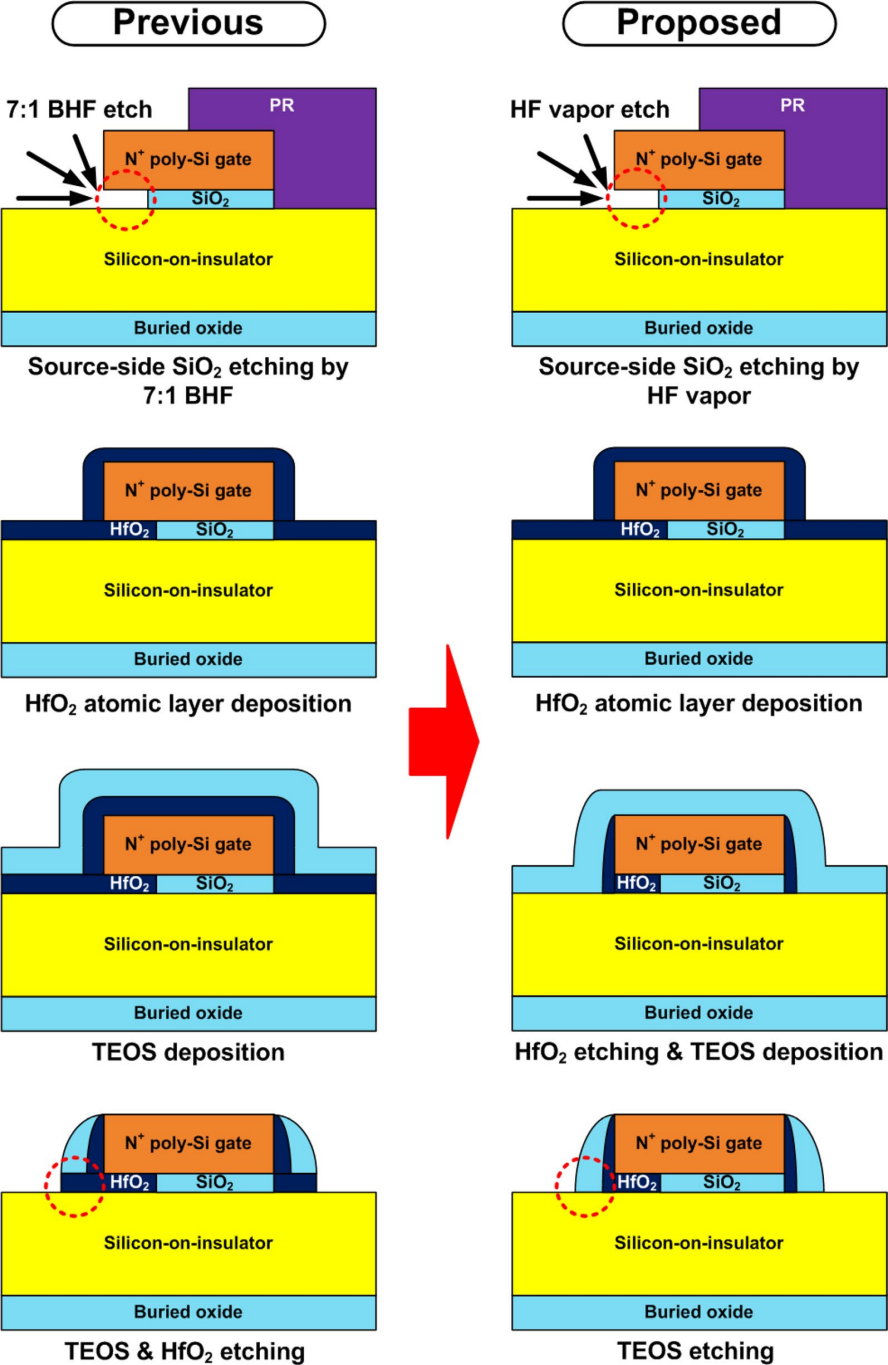
Key process proposed for performance improvement of HG TFETs

Figure 14 shows the key process flow to form HG and spacer structure of previous and proposed HG TFETs. In previous work, 7:1 BHF solution was used to etch SiO_2_ gate insulator. However, this method increased the thickness of the etched SiO_2_ gate insulator which would be filled with high-*k* material. Because BHF etched n^+^-doped polysilicon as well as SiO_2_ gate insulator, corner of the gate was also etched. As a result, thickness of HfO_2_ ($T_{HfO_{2}}$) was larger than thickness of SiO_2_ layer ($T_{SiO_{2}}$) especially at the edge of the polysilicon gate. This decreased difference of gate-to-channel coupling strength between channel regions overlapped by the high-*k* material and SiO_2_ layer which mainly determines the performance of HG TFETs. This problem has been improved by using HF vapor to etch the SiO_2_ gate insulator at the source side. HF vapor showed much better selectivity compared to 7:1 BHF solution and it enhanced thickness uniformity between *T*_HfO2_ and *T*_SiO2_. While etching the SiO_2_ insulator, the sample was held at 40 °C. It is because etch rate is too high to control and uniformity is bad when the temperature is lower than 40 °C and etch rate is too low when the temperature is higher than 40 °C.

Additionally, process for formation of the HG structure has been changed to remove the high-*k* dielectric layer on the source region. In previous work, outer TEOS spacers were formed right after HfO_2_ ALD process and then residual HfO_2_ was removed. As a result, HfO_2_ layers were remained under TEOS spacers and this decreased energy band of the source region because of increased fringe field from the gate when gate bias is applied [28]. This led to increase of *W*_tun_ and degraded performance of HG TFETs. This problem has been improved by etching HfO_2_ layers before TEOS spacers were formed. In this case, anisotropic HfO_2_ etching process should be defined to protect the HfO_2_ layer inserted under the gate. Thus, inductively coupled plasma (ICP) dry etcher was used to etch HfO_2_ layer on the source region. Adjusting etching time is very important because HfO_2_ layer on the source region should be removed and HfO_2_ layer under the gate should be protected at the same time. In addition, very careful control of HfO_2_ dry etch process was needed because silicon under the HfO_2_ layerrewis also etched well by HfO_2_ etch process condition (BCl_3_ 100 sccm, 700 W, 5 Wb, 10 mtorr). As a result, HfO_2_ layers on the source region were removed and 3-nm inner HfO_2_ spacers were remained finally.

### Device fabrication

**Fig. 15:**
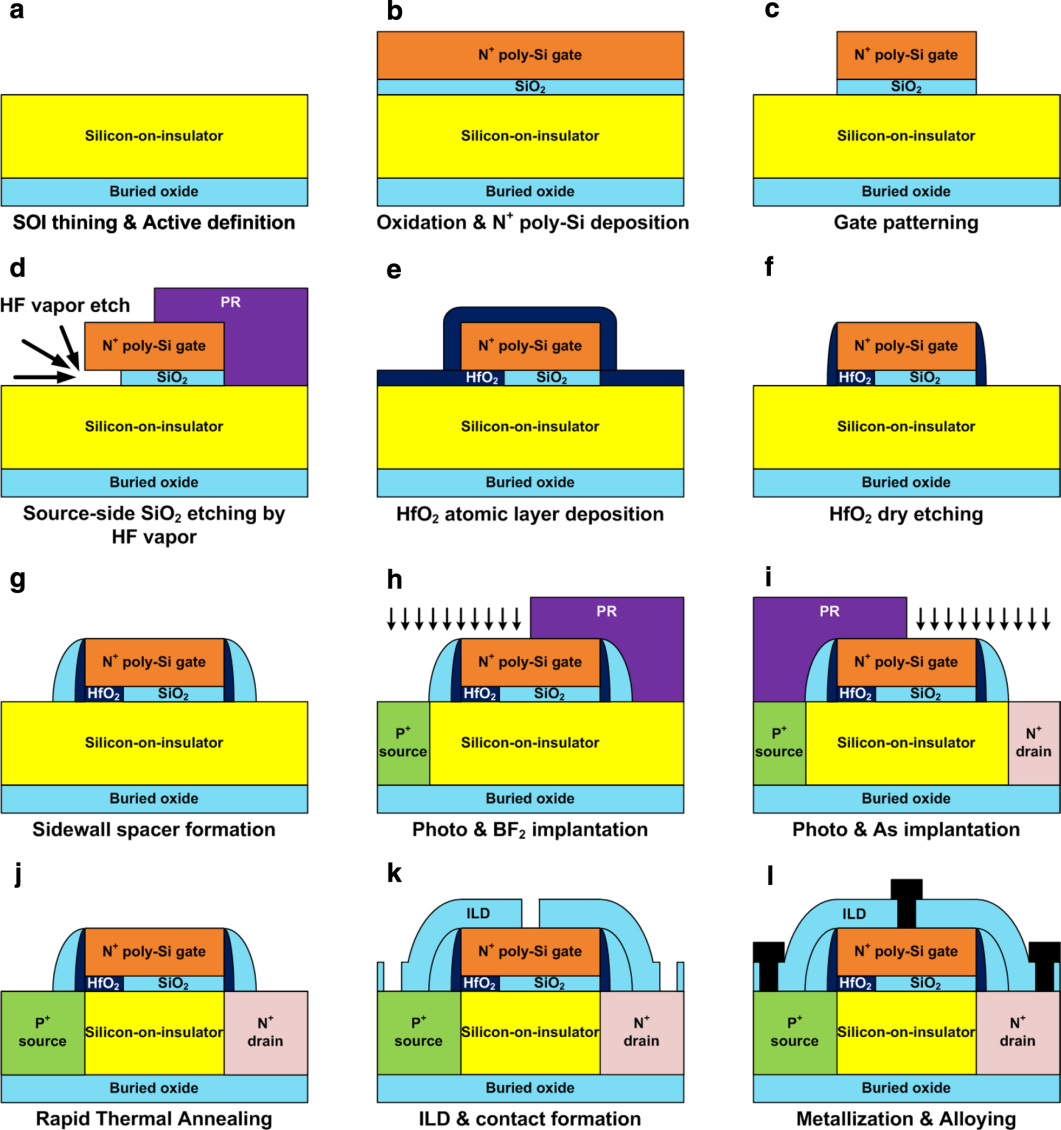
Key process for the fabrication of proposed HG TFETs

**Fig. 16:**
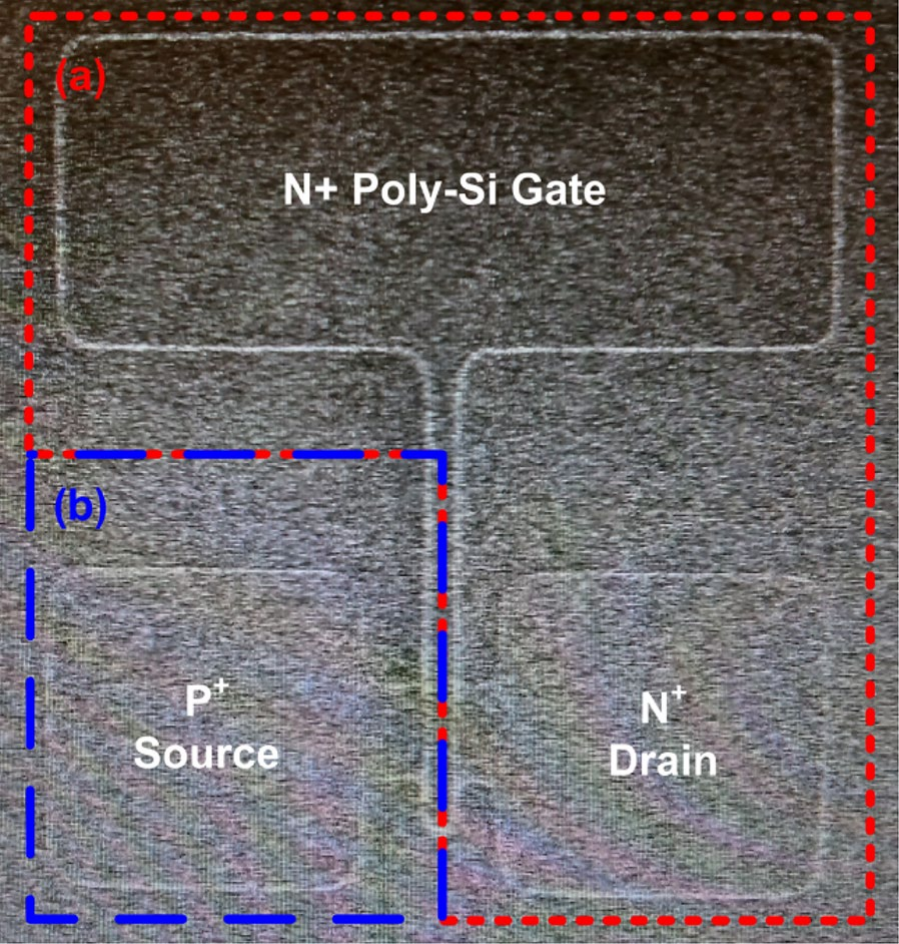
Top view SEM image of the fabricated HG TFET and masks for implantation to the **a** source and **b** drain region

In order to fabricate HG TFETs without complexity, the fabrication followed the standard CMOS process. Figure 15 shows key process flow for the fabrication of HG TFETs on SOI wafers. Most of the process steps and device structures are similar to those in previous work [27]. However, performance of fabricated HG TFETs have been improved by changing the method of etching SiO_2_ layer in Fig. 15d and changing the order of sidewall spacer formation and HfO_2_ dry etching. P-type (100) 6-inch SOI wafers (*T*_SOI_ = 100 nm and *T*_BOX_ = 375 nm) were prepared to reduce the leakage current and *T*_SOI_ was reduced to be 30 nm by thermal oxidation and removing oxide layer. Active patterns were formed on SOI substrate by photolithography and dry etching. Mesa isolation was used to separate each active region by BOX layer. The channel region is doped with p-type at 10^15^ cm^−3^. By dry oxidation and low-pressure chemical vapor deposition (LPCVD) process, the gate stack of 5-nm-thick SiO_2_ layer and 100-nm-thick phosphorus-doped polysilicon gate was formed over the active patterned substrate. The most important key process flow of HG TFETs is formation of the HG structure which is divided into two steps. First, SiO_2_ gate insulator of source side was selectively etched by using HF vapor. Before etching the SiO_2_ gate insulator only in the source side, photolithography step was performed by using mask for protecting the drain region. Second, atomic layer deposition (ALD) of 5-nm-thick HfO_2_ was performed to fill the etched gate insulator with high-*k* material. Then, HfO_2_ was etched anisotropically to remove the HfO_2_ on the gate, source and drain regions. Next, sidewall spacer was formed with deposition and etching of TEOS layer. TEOS layer was deposited using PECVD and etched by reactive ion etch (RIE). Next, asymmetric source and doping profiles were obtained by implanting different ions respectively. Compared to MOSFETs which are implemented by self-aligned source and drain ion implantation, two clear field masks for implantation to the source and the drain regions are required as shown in Fig. 16. Each mask for covering source and drain regions during implantation is described with different dotted lines. The mask for implantation to source region is the same as the one which is used when source side SiO_2_ gate insulator was selectively etched by HF vapor. Mask for implantation to the source region was designed to cover the contact area of the gate region, because gate was doped with n-type and source region was implanted with p-type. Low energy ion implantation was performed for both source and drain regions to form a steep junction profile. After photolithography for implanting source region was performed, BF_2_ ions were implanted with a dose of 1 × 10^15^ cm^−2^ at 5 keV. Following photoresist stripping, photolithography for implanting drain region was performed and As was implanted with the same condition as implantation for source region. In order to activate the dopants with minimal dopant diffusion, rapid thermal annealing (RTA) was done at 1000 °C for 3 s. As an interlayer dielectric (ILD) layer, 200-nm-thick TEOS layer was deposited by PECVD which is followed by photolithography for contact hole. Using the photoresist as a mask, ILD was etched down to the gate, source and drain regions by RIE. For a pre metal cleaning, 50:1 buffered hydrogen fluoride (BHF) solution was used for 30 s. Then, a four-level metallization (Ti-TiN-Al-TiN) was carried out in a sputtering system. Ti was used for metal adhesion, TiN was used for barrier metal, and TiN was used as an antireflection coating for photolithography of Al metal line. Metal pads were defined by photolithography and etch process. In the final step, forming gas annealing was performed at 450 °C for 30 min in H_2_/N_2_ ambient.

**Fig. 17:**
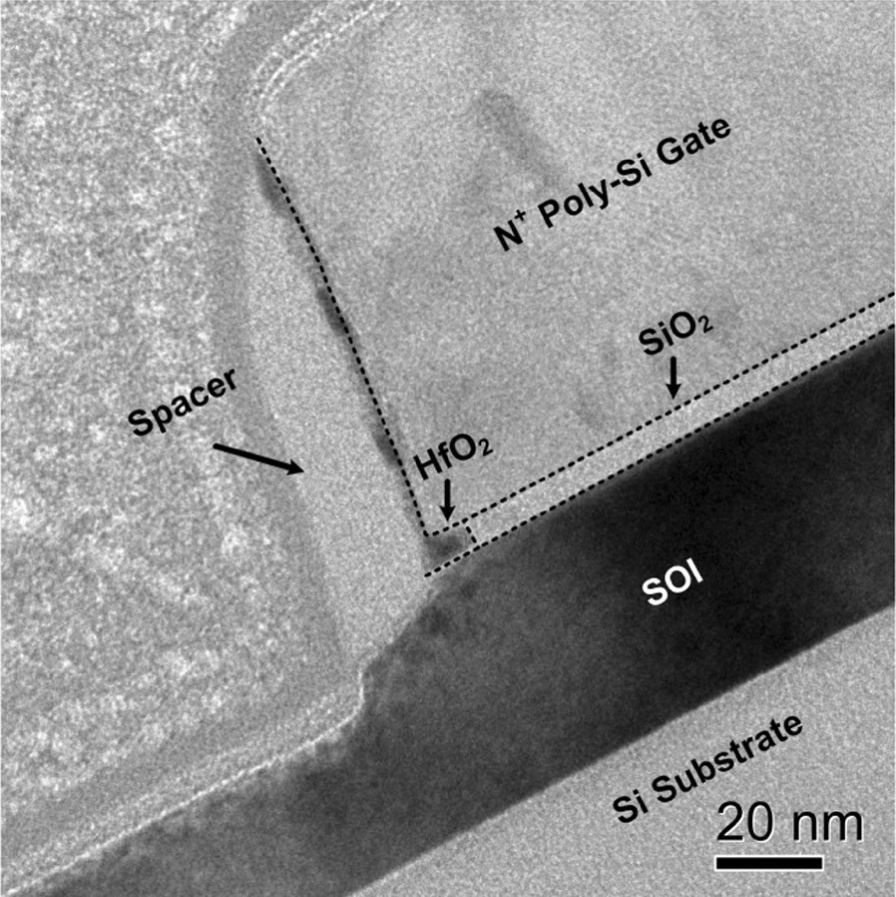
Cross-sectional TEM image of the fabricated HG TFET

Figure 16 shows the top view scanning electron microscope (SEM) image of the fabricated HG TFET. Gate length and width are 1 and 2.7 μm, respectively. Figure 17 shows the cross-sectional transmission electron microscope (TEM) image of the fabricated HG TFET. *L*_high-*k*_ of the fabricated HG TFET is ~8 nm which is similar to the optimized value [18]. Additionally, the increase of $T_{HfO_{2}}$ at the source was improved and HfO_2_ layer on the source was removed in the proposed HG TFETs as shown in Fig. 17. $T_{HfO_{2}}$ is almost equal to $T_{SiO_{2}}$. The spacers consist of 3-nm-wide inner HfO_2_ spacers and 20-nm-wide outer TEOS spacers. In order to evaluate the merits of HG TFETs, SiO_2_-only TFETs were also fabricated as control devices. Most of the process flow was the same as that of HG TFETs except for the formation of the HG structure. The SiO_2_-only TFET has only 20-nm TEOS spacers.

### Electrical characteristics

**Fig. 18:**
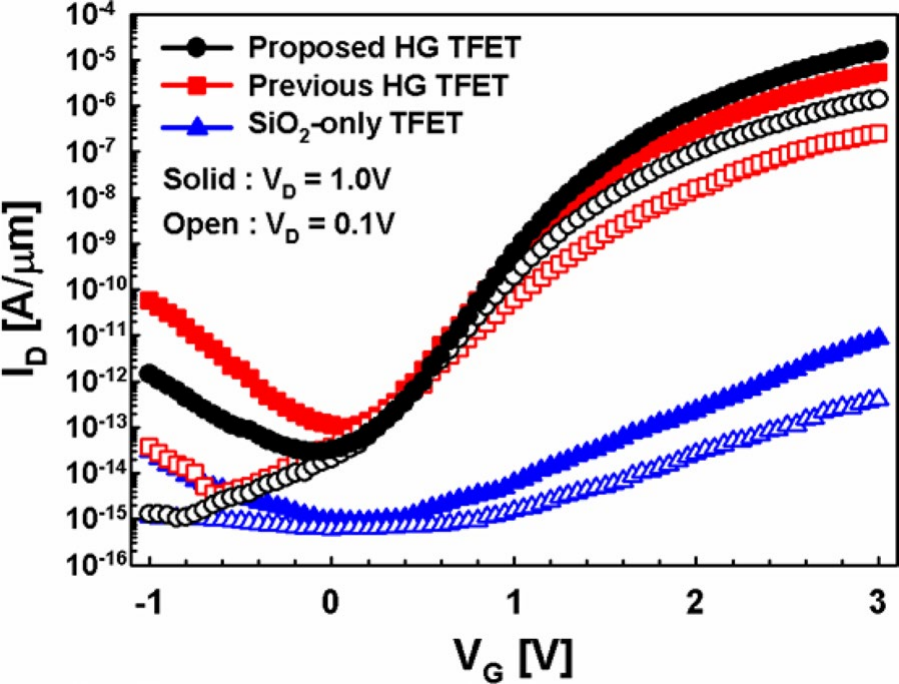
Transfer characteristics of the proposed and previous HG TFETs and SiO_2_-only TFETs

Figure 18 illustrates the transfer curves of the proposed HG TFETs compared with those of previous HG TFETs and SiO_2_-only TFETs for *V*_D_ = 0.1 and 1.0 V. Proposed HG TFETs show higher *I*_on_ and lower SS than previous HG TFETs as a result of improved device design even though *t*_ox_ is 5 nm which is larger than 3-nm *t*_ox_ of previous HG TFETs. In addition, proposed and previous HG TFETs show much higher *I*_on_ and lower SS than SiO_2_-only TFETs because HG TFETs have a local minimum of *E*_C_ resulted from locally inserted HfO_2_ at the source side gate dielectric. This reduces *W*_tun_ and increases the electric field at the tunneling junction. Though both HG and SiO_2_-only TFETs show low *I*_off_, *I*_amb_ of both kinds of devices increases as *V*_D_ increases. Especially, *I*_amb_ of HG TFETs is larger than that of SiO_2_-only TFETs because inner high-*k* spacers reduce *W*_tun_ between drain and channel region as well as *W*_tun_ between source and channel region. Thus, the underlap structure between gate and drain or reducing drain doping concentration are needed to reduce *I*_amb_ [16, 17].

**Fig. 19:**
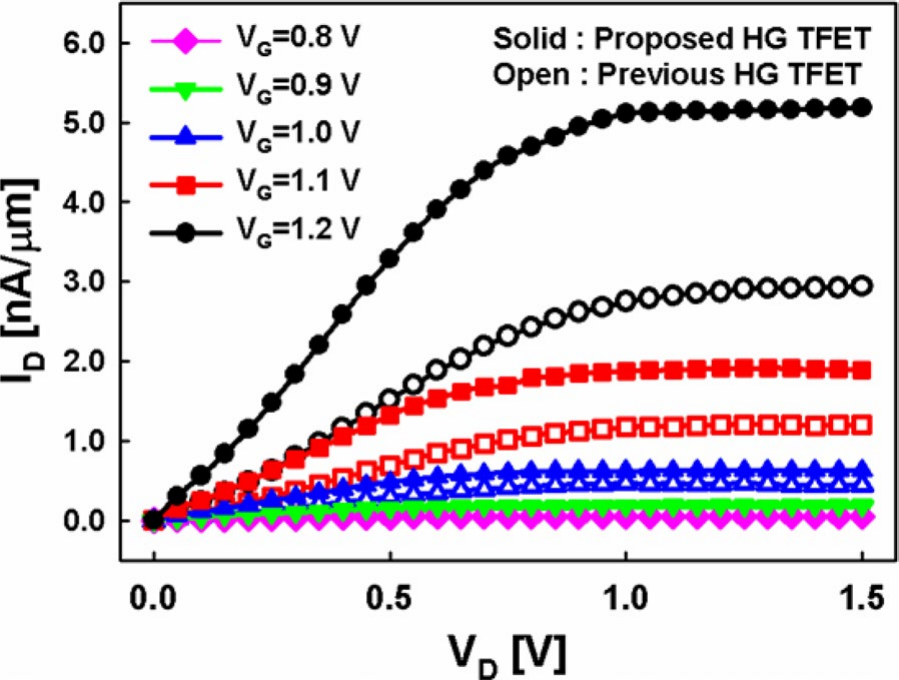
Output characteristics of the proposed and previous HG TFETs

Figure 19 shows the output characteristics of the proposed and previous HG TFETs. The output characteristics of proposed HG TFETs show better performance and lower parasitic resistance than previous HG TFETs. *I*_D_ of both kinds of HG TFETs increases with *V*_D_ slowly until it reaches its saturation value at high *V*_D_ because of high tunneling resistance. Especially, the tunneling resistance of previous HG TFETs is higher than proposed HG TFETs because *W*_tun_ is larger than proposed HG TFETs.

**Fig. 20:**
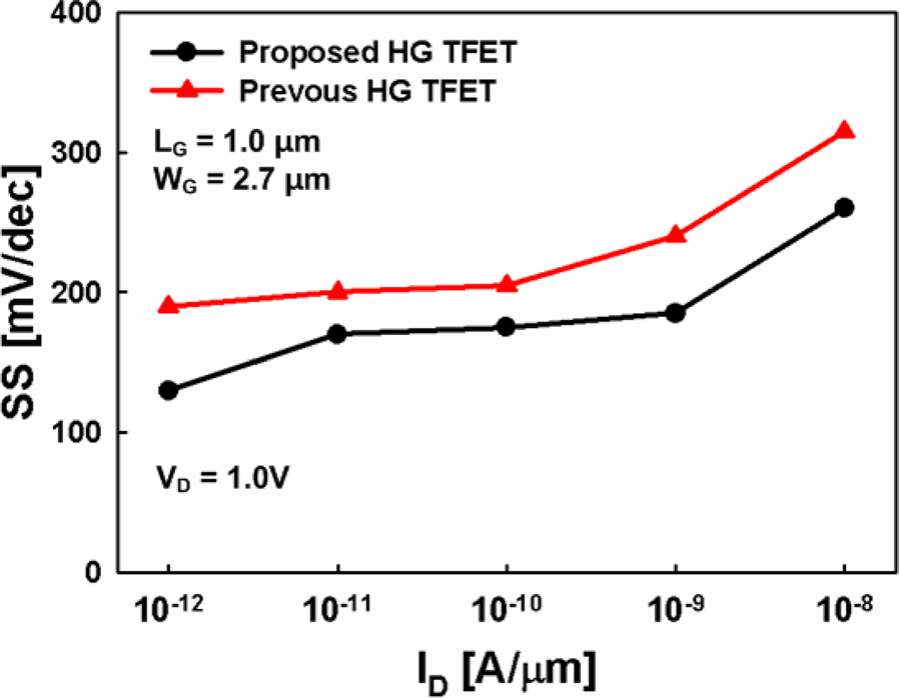
SS of the proposed and previous HG TFETs in terms of *I*_D_

Output characteristics of TFETs are different from those of MOSFETs because their mechanisms are different. While MOSFETs are saturated when the inversion layer of drain side is disappeared, most of inversion layer of TFETs is formed from the drain and surface channel potential (Ψ_s_) is pinned by *V*_D_ [30]. Thus, inversion layer formation makes *I*_D_ less sensitive to *V*_G_ and low *V*_D_ results in low Ψ_s_ which induces band-to-band tunneling currents. However, saturated currents become sensitive to *V*_G_ because *I*_D_ is determined by band-to-band tunneling without *V*_D_ influence when *I*_D_ is saturated. Figure 20 shows SS of proposed and previous HG TFETs in terms of *I*_D_. SS of SiO_2_-only TFETs is not considered because SS is much higher than those of both HG TFETs. Proposed HG TFETs show lower SS within wider current range than previous HG TFETs. Table 2 summarizes electrical characteristics of proposed HG TFET compared with those of previous HG TFET and SiO_2_-only TFET. Dimensions of proposed HG TFET are same as previous HG TFET but *T*_ox_ is different at this time. *I*_off_ is defined as *I*_D_ is equal to 1pA/μm and *V*_off_ is defined as the *V*_G_ when *I*_D_ is *I*_off_. *I*_on_ is defined as *I*_D_ when *V*_G_ is *V*_off_ + *V*_DD_. SS_min_ is minimum point swing and SS_avg_ is an average slope when *I*_D_ is from 1pA/μm to 1nA/μm. Proposed HG TFET shows higher *I*_on_ and lower *I*_min_ than previous HG TFET even though *T*_ox_ is increased. SS_min_ and SS_avg_ of proposed HG TFET are also lower than those of previous HG TFET. In addition, proposed HG TFET has an *I*_on_/*I*_off_ of 5.6 × 10^4^ at *V*_DD_ = 1 V which is comparable with other reported Si TFETs [8, 11, 12, 22].

**Table 2:** Electrical characteristics summarization of proposed HG TFET compared with previous HG TFET and SiO_2_-only TFET

	Proposed HG TFET	Previous HG TFET	SiO_2_-only TFET
*L*_G_ (μm)	1.0	1.0	1.0
*W*_G_ (μm)	2.7	2.7	2.7
*T*_ox_ (nm)	5.0	3.0	5.0
*V*_DD_ (V)	1.0	1.0	1.0
*I*_on_ (nA/μm)	56	18	0.004
*I*_min_ (pA/μm)	0.03	0.1	0.001
SS_min_ (mV/dec)	130	170	550
SS_avg_ (mV/dec)	170	200	650
*I*_on_/*I*_off_	5.6 × 10^4^	1.8 × 10^4^	3.5 × 10

Although HG TFETs are proposed for low-power application, SS of HG TFETs is larger than 60 mV/dec and current drivability is much smaller than requirements of the Low Standby Power devices [31]. First of all, abrupt doping profile is necessary for higher *I*_on_ and lower SS. Because conventional RTA is used in this work, *W*_tun_ is increased and it is difficult to control the tunneling junction. Advanced annealing methods such as spike or laser annealing can be considered for this purpose [23, 32]. In addition, tunneling current can be enhanced by using lower bandgap semiconductors such as SiGe, Ge and III-V materials [17, 19–21]. If relative permittivity of high-*k* material increases, performance of HG TFETs would be further improved.

## Conclusions

In this work, HG TFETs have been investigated through the simulation and fabrication of devices in order to demonstrate the higher performance and low-power consumption. Optimized HG TFETs showed higher *I*_on_ and, lower *I*_amb_ and SS than conventional TFETs by replacing source-side gate insulator with a high-*k* material. A high-*k* material partially located at the source side induced a local minimum of *E*_c_ due to relative permittivity discrepancy between high-*k* dielectric and SiO_2_ layer. In addition, proposed HG TFETs showed improved device performance than previous HG TFETs by improvement in device design. For the fabrication of HG TFETs with improved performance, key processes were modified. HF vapor was used to etch the source-side gate insulator uniformly and HfO_2_ etch was performed right after HfO_2_ ALD to remove the HfO_2_ layer remained on the source region. Through the electrical test of fabricated devices, proposed HG TFETs showed higher performance than previous HG TFETs and conventional TFETs in terms of *I*_on_ and SS. To sum up, it is promising that HG TFETs are alternative devices which will complement the MOSFETs for highly energy efficient ICs.
